# Supplementary material for: Rectal and Tracheal Carriage of Carbapenemase Genes and Class 1 and 2 Integrons in Patients in Neurosurgery Intensive Care Unit
Source: Antibiotics (Basel). 2022 Jul 3;11(7):886. doi: 10.3390/antibiotics11070886 (PMC9312170; doi:10.3390/antibiotics11070886)
Supplement: Supplementary file 1 [file antibiotics-11-00886-s001.zip › antibiotics-1787605-supplementary.pdf]

Table S1. Characteristics of complete genomes of *K. pneumoniae* strains

Characteristics	*K. pneumoniae* strain					
	6TKP/19b	4PKP/19c	6PKP/19c	7TKP/19c	8TKP/19c	15PKP/19c
Strain ID	SCPM-O-B-8913	SCPM-O-B-8914	SCPM-O-B-8916	SCPM-O-B-8917	SCPM-O-B-8918	SCPM-O-B-8921
ST/CT	ST29/K23	ST39/K23	ST39/K23	ST39/K23	ST39/K23	ST14/64/K147
Source	tracheal swab	rectal swab	rectal swab	tracheal swab	tracheal swab	rectal swab
Isolation date	24-Oct-2019	21-Oct-2019	21-Oct-2019	21-Oct-2019	21-Oct-2019	21-Oct-2019
WGS features						
BioSample ID	SAMN18878034	SAMN18878035	SAMN18878037	SAMN18878038	SAMN18878039	SAMN18928739
Read Archive	SRR14338903	SRR14338902	SRR14338900	SRR14338899	SRR14338898	SRR14493698
GenBank	JAGUTU000000000	JAGUTT000000000	JAGUTS000000000	JAGUTR000000000	JAGUTQ000000000	JAHAVN000000000
GC-content, %	57.09	56.71	56.82	56.61	56.75	57.05
Reads	6743247	2144028	717966	891800	610484	15382567
Contigs	178	223	422	274	291	94
Genome Size, bp	5604320	5988366	5716153	5960613	5895045	5637725
Read Depth, ×	194	194	256	260	261	195
Coverage, ×	250	78	32	38	26	566
N50 value, bp	301137	190681	41652	99187	93206	224193
Genes	5573	6013	5899	6025	5970	5577
Antimicrobial resistance genetic determinants						
Aminoglycoside	*aac(6′)*<br>*aadA2*<br>*aph(3′′)*<br>*aph(6)*	*aac(6′)*<br>*aac(3)-IIa*<br>*ant(3′′)-Ia*<br>*ant(2′′)-Ia*<br>*aadA2*<br>*armA*<br>*aadA8b*	*aac(6′) aac(3)-IIa*<br>*aadA1*<br>*aadA2*<br>*armA*	*aac(6′) aac(3)-IIa*<br>*ant(2′′)-Ia*<br>*aadA1*<br>*armA*	*aac(6′) aac(3)-IIa*<br>*ant(3′′)-Ia*<br>*ant(2′′)-Ia*<br>*aadA2*<br>*armA*	*aac(6′)*<br>*aadA1*<br>*aadA2*<br>*armA*<br>*aph(3′)*<br>*aph(3′′)*<br>*aph(6)*
Fosfomycin	*fosA*	*fosA*	*fosA*	*fosA*	*fosA*	*fosA*
Phenicol	*catA1*<br>*catB3*	*cmlA1*<br>*catA1*<br>*catB3*	*cmlA1*<br>*catA1*<br>*catB3*	*cmlA1*<br>*catA1*<br>*catB3*	*cmlA1*<br>*catA1*<br>*catB3*	*catB3*
Quinolone	*qnrS1*<br>*oqxA*<br>*oqxB*	*qnrS1*<br>*oqxA*<br>*oqxB*	*qnrS1*<br>*oqxA*<br>*oqxB*	*qnrS1*<br>*oqxA*<br>*oqxB*	*qnrS1*<br>*oqxA*<br>*oqxB*	*qnrS1*<br>*oqxA*<br>*oqxB*
Sulfonamide	*sul1*<br>*sul2*	*sul1*	*sul1*	*sul1*	*sul1*	*sul1*<br>*sul2*
Trimethoprim	*dfrA1*<br>*dfrA12*<br>*dfrA14*	*dfrA12*	*dfrA12*	*dfrA12*	*dfrA12*	*dfrA12*
Macrolide	-	*mphE*<br>*msrE*	*mphE*<br>*msrE*	*mphE*<br>*msrE*	*mphE*<br>*msrE*	*mphE*<br>*msrE*
Virulence genetic determinants (locuses)						
Type 3 adhesin	*mrk*	*mrk*	*mrk*	*mrk*	*mrk*	*mrk*
Yersiniabactin	*irp*	*irp*	*irp*	*irp*	*irp*	-
Yersiniabactin transcriptional regulator	*ybt*	*ybt*	*ybt*	*ybt*	*ybt*	-
Siderophore yersiniabactin receptor	*fyu*	*fyu*	*fyu*	*fyu*	*fyu*	-
Aerobactin siderophore synthesis	-	*iuc*	-	*iuc*	*iuc*	-
Ferric aerobactin receptor	-	*iut*	-	*iut*	*iut*	-
Plasmids and Molecular systems protecting bacteria from foreign DNA						
Plasmids	ColRNAI<br>IncL/M<br>IncFIB<br>IncFII	ColRNAI<br>IncA/C2<br>IncFIB<br>IncFII<br>IncHI1B	ColRNAI<br>IncC<br>IncFIB<br>IncFII<br>IncHI1B	ColRNAI<br>IncC<br>IncFIB IncFII<br>IncHI1B	ColRNAI<br>IncA/C2<br>IncFIB<br>IncFII<br>IncHI1B	ColRNAI<br>IncFIB<br>IncFIA<br>IncFII IncHI1B
Type I Restriction Enzymes	-	M.Kpn928I<br>S.Kpn1420II	M.Kpn928I<br>S.Kpn1420II	M.Kpn928I<br>S.Kpn1420II	M.Kpn928I<br>S.Kpn1420II	M.Kpn928I StySKI<br>M.Sen1921IM.EcoJA03PI<br>S.Kpn1420II
Type II Restriction Enzymes	M.EcoRII Eco128I<br>M.Kpn34618Dcm	M.Kpn34618Dcm	M.Kpn34618Dcm	M.Kpn34618Dcm	M.Kpn34618Dcm	M.Kpn34618Dcm
CRISPR-Cas	No	Type I, TypeU	Type I	Type I, TypeU	Type I, TypeU	Type I
